# Cancer Recurrence in Operated Primary Oral Squamous Cell Carcinoma Patients Seems to Be Independent of the Currently Available Postoperative Therapeutic Approach: A Retrospective Clinical Study

**DOI:** 10.3390/curroncol32040208

**Published:** 2025-04-01

**Authors:** Shahram Ghanaati, Samuel Ebele Udeabor, Anne Winter, Robert Sader, Anja Heselich

**Affiliations:** 1 Department of Oral, Cranio-Maxillofacial, and Facial Plastic Surgery, Johann Wolfgang Goethe University, 60596 Frankfurt am Main, Germany; anne.winter@unimedizin-ffm.de (A.W.); r.sader@em.uni-frankfurt.de (R.S.);; 2 Department of Oral and Maxillofacial Surgery, College of Dentistry, King Khalid University, Abha 61421, Saudi Arabia; seudeabor@kku.edu.sa

**Keywords:** oral squamous cell carcinoma, cancer recurrence, adjuvant therapy, tumor margins, frozen section

## Abstract

Despite advances in treatment, recurrence rates in oral squamous cell carcinoma (OSCC) remain high. Prognostic outcomes vary in terms of local recurrence, metastasis, and overall survival. A retrospective cohort analysis was conducted on OSCC patients who underwent primary surgery at the Department of Craniomaxillofacial and Facial Plastic Surgery, University Medical Center Frankfurt, between January 2014 and December 2020. Demographic data, tumor characteristics, surgical details, intraoperative frozen section results, and recurrence patterns were first assessed for availability. Subsequently, the available data relevant to each endpoint were analyzed. A total of 169 patients were analyzed (mean age: 64 years). The tongue was the most affected site and had the highest recurrence rate, followed by the floor of the mouth. Overall, 24.3% of patients experienced recurrence, with most cases occurring within the first year. T2 tumors had the highest recurrence rates. Between patients with and without adjuvant therapy, recurrence rates were comparable. Positive surgical margins were more common in recurrence cases, but no significant correlation was found between margin status and recurrence in patients without adjuvant therapy. Based on the analyzed data, achieving recurrence-free survival in OSCC does not solely depend on surgical technique or adjuvant therapy. Instead, early recognition of individual tumor characteristics and even tumor biology should guide personalized treatment planning. Notably, tumors of the tongue and floor of the mouth exhibited high recurrence rates regardless of disease stage, raising the question of whether primary chemoradiotherapy (CRT) could achieve better outcomes than surgery. Further studies are needed to evaluate the role of CRT as a first-line treatment for OSCC in these locations.

## 1. Introduction

Oral squamous cell carcinoma (OSCC) is a subset of head and neck squamous cell carcinoma and accounts for more than 90% of all oral cancers [1,2,3]. Globally, oral cancers constitute approximately 5% of reported cancer cases and 8% of cancer-related deaths annually [2]. Surgery, chemotherapy, and radiotherapy are the main treatment options. These can be used separately or in combination depending on the stage of the disease and spread. However, cancer treatment generally has been evolving over the years and new modalities are regularly being researched to augment these traditional options of surgery, chemotherapy, and radiotherapy [3].

The achievement of local recurrence-free disease and improved overall survival are the primary targets in the management of OSCC patients. These outcomes are, however, influenced by a host of factors ranging from the stage of the disease, nodal involvement, extracapsular spread, perineural invasion, type of surgery, tumor margin status at surgery, and the use of adjuvant therapy, among others [4].

Achieving a negative intraoperative margin, especially via frozen section, is deemed a very important step toward preventing oral cancer recurrence and improving overall survival [4]. The histological diagnosis of margins is based on guidelines issued by the Royal College of Pathologists [5] with margins divided into involved (<1 mm), close (1–5 mm), or clear (>5 mm). The margin status is important in predicting the risks of recurrence and death [6] and is, therefore, considered an essential factor in the decision-making for adjuvant treatment options [4,7,8]. Additionally, local and regional recurrences with distance metastasis and overall survival in OSCC patients have all been shown to be negatively influenced by inadequate intraoperative resection margins [7]. Some authors, therefore, are of the opinion that a negative intraoperative surgical margin (R0) with frozen section is among the strongest predictors of local recurrence-free disease outcome and consequently disease-free survival [4,8,9] and thus advocate for even more “judicious” use of frozen section in OSCC management [8].

On the contrary, Pathak et al. [10], in their review of 416 cases of oral cancer patients who were surgically treated in a comprehensive cancer center, reported that margin evaluation using intraoperative frozen section did not independently affect the disease outcome in terms of local control and survival. Similarly, another study [10] also found out that surgical excision of low T-stage (T1 and T2) buccal mucosa cancers, even with negative margins, still resulted in a high rate of local recurrences, leading the authors to suggest that even early tumors in this category may require adjuvant treatment to achieve local control [11].

Despite these conflicting results, it should be noted that many other factors apart from surgical margins play crucial roles in determining the outcome of OSCC management. Among these factors is adjuvant therapy, which comprises mainly radiotherapy, chemotherapy, and, in some cases, immunotherapy.

The decision to use adjuvant therapy post-surgery in our center is made by the tumor board expert panel with the patient’s consent. Each case is considered individually depending mainly on the stage of the disease at presentation, nodal involvement, extracapsular nodal extension, perineural invasion, intraoperative surgical margin status, and post-surgical histopathological outcome. Generally, adjuvant therapy is usually reserved for stage III/IV resectable cases of OSCC with perceived high-risk factors for recurrence and poor prognosis. These high-risk factors include positive surgical margins, extracapsular spread, multiple cervical lymph node metastasis (level IV or level V), perineural invasion, and vascular tumor embolism [11].

The adjuvant therapy is believed to help in achieving loco-regional control and improving overall survival. Some authors advocate its use even in early cases of some categories of OSCC, citing a high rate of recurrence with only local surgical excision [12]. However, a recent multicenter prospective study [13] concluded that adjuvant therapy in early OSCC (TI/T2 with single ipsilateral cervical node pN1) affected only the time of disease progression but did not seem to affect the overall survival. In the recently published INVERT trial, the feasibility and predictive factors of response to neoadjuvant chemoradiotherapy (nCRT) in locally advanced oral cavity cancer (LA-OCC) were investigated. The interim analysis showed a pathological complete response (pCR) in 41% of patients. This study’s proposed predictive factors such as MRI signal changes and tumor-infiltrating immune cells correlating with the treatment response. The authors conclude that the treatment regimen starting with nCRT, followed by radical surgery, is feasible and achieves high response rates [14]. This dichotomy of opinions and results documented over the years from the management of OSCC, therefore, makes it difficult to predict the outcome of OSCC in terms of local/regional recurrence, metastasis, and overall survival. This then raises the question as to what is truly responsible for the poor prognosis following OSCC management, even in cases of early-stage disease where the aforementioned high-risk factors were non-existent. Is the outcome primarily dependent on surgical performance and resection margins, or the chosen adjuvant therapy, as is widely believed? Or is there no such dependency, with factors like the biological nature of oral cancer potentially being the missing link that influences recurrences and survival, despite the techniques and tools used in its primary management?

The aim of this retrospective study was, therefore, to analyze the management of an OSCC patient cohort, with a particular focus on the influence of surgical margins and adjuvant therapy on local recurrence and overall survival.

## 2. Material and Methods

### 2.1. Study Design and Patient Cohort

A retrospective analysis of patients with histopathological proof of oral squamous cell carcinoma (OSCC) and who had their primary surgery in the Department of Craniomaxillofacial and Plastic Surgery, University Medical Center Frankfurt, Germany, from 1 January 2014 to 31 December 2020, was conducted. Patients were identified by analyzing internal databases and confirmed manually.

#### 2.1.1. Inclusion and Exclusion Criteria

Inclusion criteria were histologically diagnosed intraoral OSCC (lip to retromolar region), operable tumor with no history of previous surgery, comparable surgical approach regarding tumor resection regime, indicated for frozen sections, and available follow-up data for overall and recurrence-free survival. Exclusion criteria were the presence of other malignancies, extraoral localization, and primary nonsurgical intervention (e.g., chemoradiotherapy (CRT)).

#### 2.1.2. Therapeutic Approach

**Figure 1:**
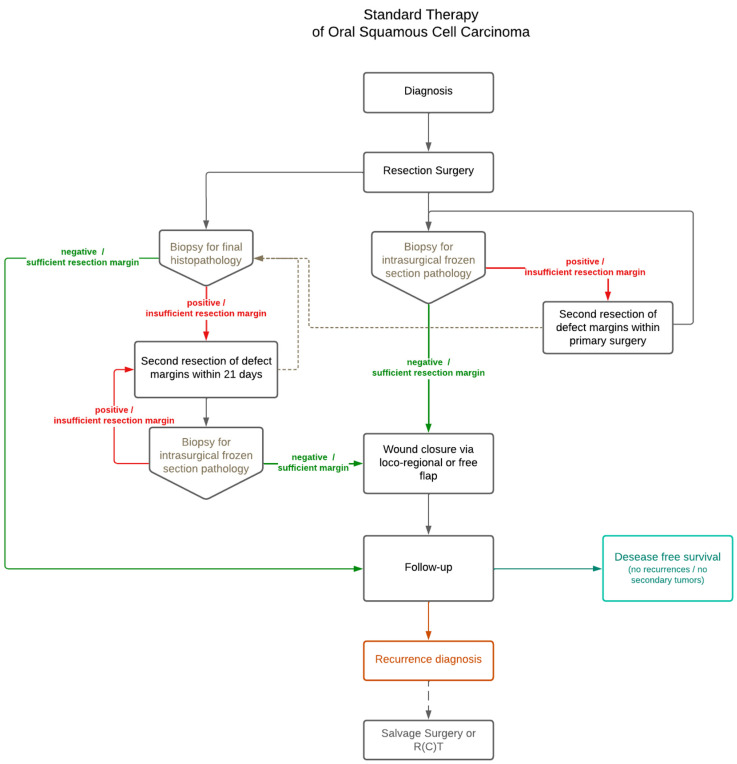
Flowchart of the usual therapeutic approach for oral squamous cell carcinoma, as implemented in the study center and in accordance with standard clinical guidelines.

OSCC patient’s therapeutic regime of included patients was in accordance with the usual therapeutic approach at the Department of Craniomaxillofacial and Facial Plastic Surgery, University Medical Center Frankfurt, Germany, and according to the corresponding guidelines for OSCC indications (see Figure 1). Therapy planning was further based on the individual patient’s clinical history and consultation by the tumor board expert panel. In summary, the usual approach after histopathological confirmation of OSCC was surgical resection of the tumor including sufficient safety margin to reach R0 status. If intraoperatively obtained biopsies analyzed as frozen sections or post-surgical histopathological evaluation of all taken biopsies revealed residual OSCC within samples, a second tumor resection of the compromised area was performed. The same approach was chosen if the resection margin had not yet reached R0. Depending on the histopathological outcome and consent achieved by the tumor board expert panel, adjuvant therapy could be indicated post-surgery. Application of adjuvant therapy (radiotherapy, chemotherapy, or chemoradiotherapy) was done at the corresponding specialized departments at the University Medical Center, Frankfurt, Germany. All included patients were observed post-surgery in a defined follow-up regime at the Department of Craniomaxillofacial and Facial Plastic Surgery, including clinical and radiological evaluation of the healing progress and disease-free survival.

Patients diagnosed with recurrence during follow-up were presented to the tumor board expert panel, and a therapy plan was discussed and planned. Depending on the individual diagnosis, surgery with or without adjuvant therapy was planned. If surgical intervention was not possible due to the patient’s individual situation, alternative approaches, e.g., radio-/chemoradiotherapy, were planned, or, if necessary, palliative care was organized.

#### 2.1.3. Ethical Approval

This retrospective study was approved by the Institutional Review Board of the Ethical Committee of the Medical Department of Goethe University (IRB approvals #03/2013; #40/18; #2021–76).

### 2.2. Screening and Data Evaluation

**Figure 2:**
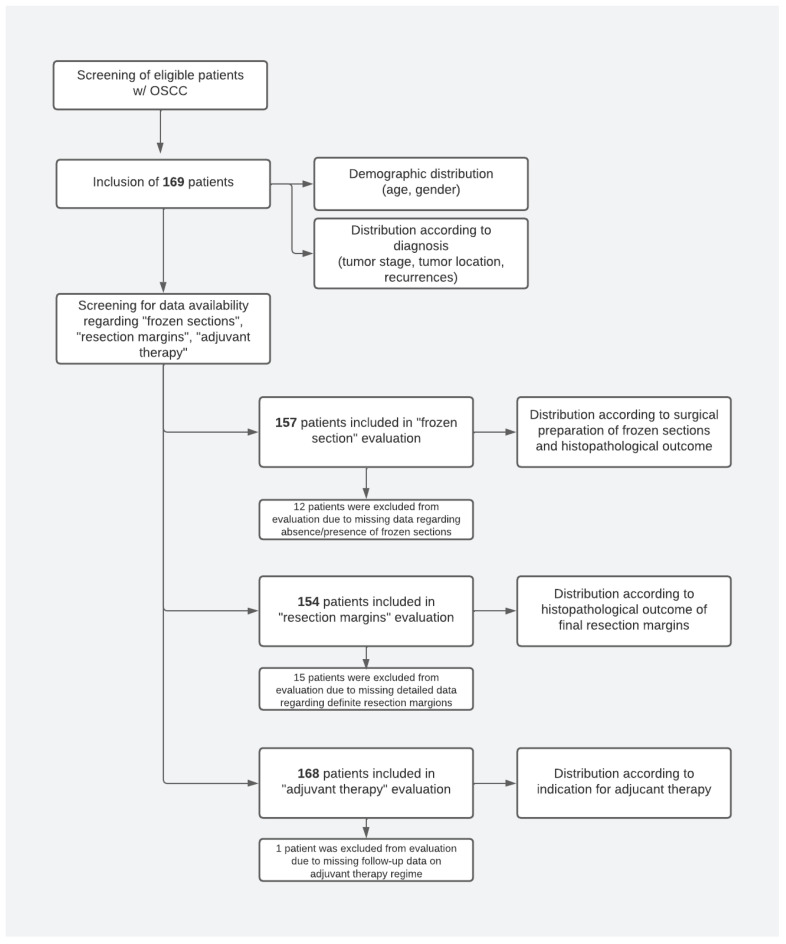
Patient screening and patient distribution to evaluated endpoints.

Patients were screened for eligibility for inclusion according to tumor location and clinical approach (see Figure 2). The following parameters were recorded and evaluated:

- Demographic parameters: age and gender.
- Tumor-related parameters: anatomical localization of the tumor, T-classification, and presence/absence of recurrences.
- Therapy regime-related parameters: adjuvant treatment (presence/absence) and histopathological outcome of frozen sections, further resections, and histopathological outcome of final resection margins.

### 2.3. Statistics

The descriptive statistics and statistical analysis were calculated using GraphPad Prism Analysis software (version 10.1.1, GraphPad Software, LLC., San Diego, CA, USA). Statistical analysis of nominal data was performed using the two-sided Fisher’s exact test at a confidence interval of 95%. Statistical analysis of age distributions according to sex and/or T-stage was performed using ordinary one-way ANOVA with a post hoc test for multiple comparisons (Tukey) at a confidence interval of 95%.

## 3. Results

### 3.1. Included Patients and Data Availability

A total of 169 OSCC patients who met the inclusion criteria were included in this study (Figure 2). Data availability for analysis of demographic data, tumor distribution, recurrences, adjuvant therapy, and surgical endpoints, including frozen sections and resection margins, was assessed.

Demographic information, as well as information on tumor site, tumor stage, and recurrences, was available for 169 patients. Therefore, 169 patients were included in the analysis of the respective endpoints.

Data regarding the absence/presence of frozen sections taken during surgery and the corresponding histopathological outcome were available for 157 patients. A total of 12 patients had to be excluded from the evaluation related to frozen sections due to missing data on either the absence/presence of frozen sections or missing data on the histopathological outcome in patients where frozen sections had been taken.

Detailed data regarding final histopathological resection margins necessary for categorizing patients according to the Royal College of Pathologists (RCPath) 1998 [11] were available for 154 patients. A total of 15 patients had to be excluded from the evaluation of histopathological resection margins due to missing/insufficient data on the definite distances of OSCC residuals to resection margins.

Sufficient information for evaluation regarding adjuvant therapy was available for 168 of 169 included patients. One patient had to be excluded due to insufficient information on the adjuvant therapy regime in follow-up (Figure 2).

### 3.2. Evaluation of Patient Distribution

#### 3.2.1. Oral Squamous Cell Carcinoma Sites

The site of OSCC in the total cohort of *n* = 169 patients was found in six different intraoral regions: lip (1.78%), lower jaw (16.0%), upper jaw (9.47%), tongue (33.14%), floor of the mouth (30.18%), palate (4.73%), and buccal mucosa (4.73%) (see Table 1).

#### 3.2.2. Demographics and T-Stage

**Figure 3:**
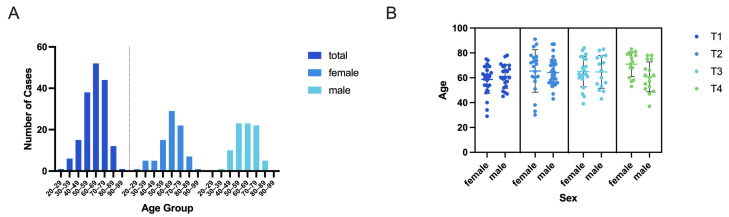
Age distribution. (**A**) Gaussian distribution within age groups of total patients included, and sub-distribution according to sex. (**B**) Evaluation of mean age according to sex and in relation to tumor stage. Data are represented as total numbers (**A**) or as a scatter plot with mean ± SD (**B**); n_total_ = 169 patients.

The analyzed patient cohort (*n* = 169) showed an equal distribution of patients according to gender, consisting of 85 female and 84 male patients (Table 2). The mean age of the analyzed patients was 63.99 ± 13.37 years for female patients and 62.82 ± 11.10 years for male patients. Distribution according to age groups showed a Gaussian distribution with the maximum peak at the age group of 60–69 years for the total patient cohort, as well as for both sexes if plotted individually (see Figure 3A).

OSCC of all T-stages, T1 to T4, was found in the analyzed patient cohort, with 30.18% (17.13% female, 13.02% male) T1 tumors, 30.18% (11.84% female, 18.34% male) T2 tumors, 21.30% (12.43% female, 8.87% male) T3 tumors, and 18.34% (8.87% female, 9.47% male) T4 tumors (see Table 2).

The mean age of female and male patients was comparable throughout the patient cohort, without a significant difference according to gender within each T-stage group (see Figure 3B). The mean age of female patients was 58.66 ± 11.14 for patients with T1 tumors, 65.45 ± 17.06 for T2-tumor patients, 65.00 ± 12.31 for T3-tumor patients, and 70.93 ± 9.85 for T4-tumor patients. The mean age of male patients was comparable at 61.09±9.57 years for patients with T1 tumors, 64.19 ± 10.73 years for T2-tumor patients, 64.73 ± 13.22 years for T3-tumor patients, and 60.75 ± 12.01 years for T4-patients.

#### 3.2.3. Recurrences

- Overall Recurrence Distribution and Occurrence of Recurrences Within Follow-Up

Patients’ follow-up revealed diagnosed recurrence in 30.8% of female patients and 33.3% of male patients (see Table 3). The majority of recurrences were diagnosed within the first year (68.3% of all recurrences), whereas less than 1/3 of the recurrences were observed within the second (4.9%) or third (7.3%) year of follow-up, or even later (19.5%) (Table 4). The probability for recurrence based on gender was not statistically significant, neither for total numbers of patients nor for sub-analysis according to the year of recurrence diagnosis (see Table 4).

The overall distribution of patients by T-stage was comparable to the distribution of recurrences (see Table 5). Among patients without recurrences, the T-stage distribution was as follows: T1: 79.3% (female)/81.8% (male), T2: 60.0% (female)/67.7% (male), T3: 85.7% (female)/73.3% (male), and T4: 80.0% (female)/81.3% (male).

For patients with recurrences within the first year, the distribution was as follows: T1: 10.3% (female)/9.1% (male), T2: 25.0% (female)/22.6% (male), T3: 14.3% (female)/20.0% (male), and T4: 13.3% (female)/18.8% (male).

Finally, for patients with recurrences occurring later than the first year, the distribution was as follows: T1: 10.3% (female)/9.1% (male), T2: 15.0% (female)/9.7% (male), T3: 0% (female)/6.7% (male), and T4: 6.7% (female)/0% (male).

- Recurrence Distribution According to Tumor Localization

**Figure 4:**
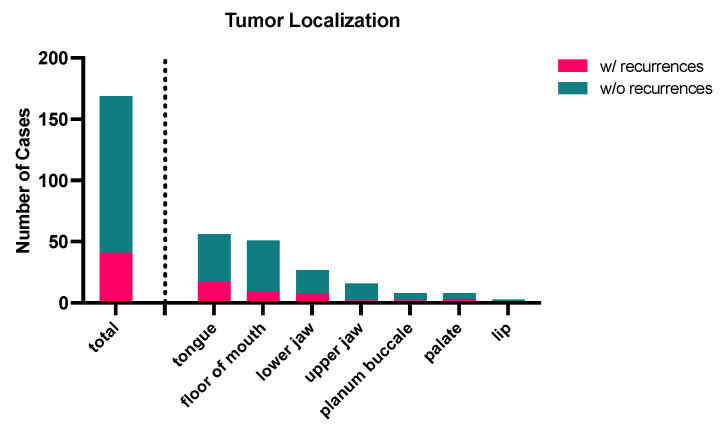
Tumor distribution according to localization. OSCC was found in seven different locations, with most of the cases found in the tongue and the floor of the mouth, followed by the lower and upper jaw. Only a small portion of cases was found in the buccal mucosa (planum buccale), palate, or lip. Independent of the location, most of the cases did not show recurrences (green). Less than 25% of total cases were diagnosed with recurrence (pink) during follow-up. Most cases were found, as expected, in locations with the highest incidences: tongue, floor of mouth, and lower jaw. Data are represented as absolute numbers of cases (n_total_ = 169 patients).

The overall ratio of patients with recurrences was 24.3% compared to 75.7% of patients without recurrence diagnosis. As expected, the highest number of cases with recurrences was found in the locations with the highest OSCC frequency, such as the tongue, floor of mouth, and lower jaw. Lower numbers were found for locations with a lower overall number of total cases within the analyzed patient cohort (see Figure 4). The ratio of cases w/recurrences showed the following distribution: 43.6% for tumors in the tongue (17 out of 56 cases), 21.4% for tumors in the floor of the mouth (9 out of 51 cases), 29.6% for tumors in the lower jaw (8 out of 27 cases), 12.5% for tumors in the upper jaw (2 out of 16 cases), 25% for tumors in the buccal mucosa (2 out of 8 cases), and 37.5% for tumors at the palate (3 out of 6 cases). No recurrence cases were observed in the three OSCC patients with tumors located on the lip.

### 3.3. Recurrence-Dependent Survival

**Figure 5:**
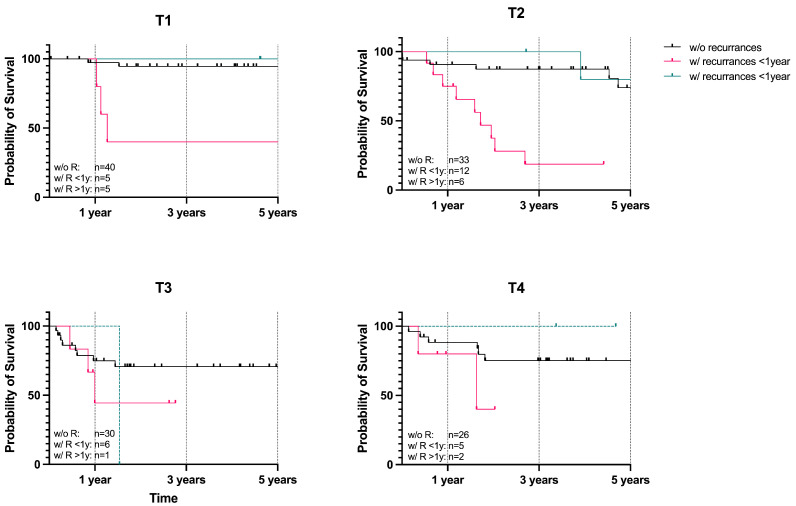
Kaplan–Meier survival analysis of OSCC patients with T1, T2, T3, and T4 tumors, without recurrences, with recurrences within the first year, and with recurrences after one year. OSCC patients without recurrences show a clear T-stage dependent survival, with increasing risk as the stage advances, with T3 as the only exception, showing lower survival probability than T4. OSCC patients with recurrences within the first year (pink lines) exhibit a lower survival probability compared to patients without recurrences (black lines). Within the first year, the survival rate remains T-stage dependent, but this dependency diminishes in the following years. OSCC patients with recurrences diagnosed after the first year post-primary surgery (green lines) show a better survival probability, with only T3 patients at high risk; however, these findings should be interpreted with caution due to the low number of cases (T1: *n* = 50, T2: *n* = 51, T3: *n* = 37, T4 *n* = 33).

To assess whether the occurrence of recurrences, as well as tumor stage, influences survival, a Kaplan–Meier survival analysis was conducted for patients without recurrences, with recurrences within the first year, and with recurrences occurring after one year. The analysis revealed that OSCC patients without recurrences demonstrated T-stage-dependent survival, with the risk of poor survival increasing as the stage advanced, except for T4 tumors, which showed higher survival rates than T3 tumors (Figure 5, black lines). T1 patients had a survival probability of 97.30% after 1 year and 94.52% after 3 and 5 years; T2 patients had 90.81% after 1 year, 87.45% after 3 years, and 73.99% after 5 years; T3 patients had 74.92% after 1 year and 70.54% after 3 and 5 years; and T4 patients had 84.09% after 1 year and 75.24% after 3 and 5 years.

Patients with recurrences within the first year showed a significantly lower survival probability compared to those without recurrences. T-stage dependency was evident during the first year, but this dependency diminished in the following years (Figure 5, pink lines). T1 patients had a survival probability of 80.0% after 1 year, 40.0% after 3 years, and 40.0% after 5 years; T2 patients had 75.0% after 1 year and 18.75% after 3 years; T3 patients had 44.44% after 1 year and 44.44% after 2.5 years; and T4 patients had 80.0% after 1 year and 40.0% after 2 years.

For patients with recurrences diagnosed after the first year post-primary surgery, the survival probability was better, although T3 patients still faced a higher risk (Figure 5, green lines). However, the results for recurrences occurring after one year should be interpreted with caution due to the low number of cases. T1 patients had a survival probability of 100.0% after 1, 3, and 5 years; T2 patients had 100.0% after 1 year and 80.0% after 3 and 5 years; T3 patients had 0% after 1 year; and T4 patients had 100.0% after 1, 3, and 4.5 years.

### 3.4. Adjuvant Therapy Regime in OSCC Patients with and Without Recurrences

The therapy regime of OSCC patients can include adjuvant therapy, either radiotherapy, chemoradiotherapy, or chemotherapy. Indication for adjuvant therapy is based on the individual patient’s clinical history and consultation by the tumor board expert panel. Information on adjuvant therapy was available for *n* = 168 of 169 patients (see Figure 2). The percentage of patients diagnosed with recurrence who had additional adjuvant therapy (24.05%) was comparable to that of recurrence patients w/o adjuvant therapy (24.72%) (see Table 6). Statistical analysis showed no increased probability of recurrence if no adjuvant therapy was given (*p* > 0.999).

Sub-analysis of patients who received adjuvant therapy showed that 26.67% of patients undergoing chemoradiotherapy (CRT) and 36.67% of patients receiving radiotherapy developed recurrence during follow-up (see Table 7).

### 3.5. Histopathological Outcomes

#### 3.5.1. Intrasurgical Histopathological Evaluated Frozen Sections

After the primary resection of OSCC tumors, preparation of further tissue samples from the resection margin for immediate histopathological analysis and verification of complete resection is usually performed. Histopathological analysis of these tissue samples is performed on so-called frozen sections. If frozen sections were taken, depending on their histopathological outcome, re-excision of the critical region was indicated when hints for residuals of the primary OSCC tumor were given (see Figure 1). If residual OSCC was found in the frozen section, it was defined as “positive”. Exact margin details, as reported in the final histopathology (e.g., close, involved), were not provided here. Samples without residual OSCC were defined as “negative”.

- Follow-Up Therapy of Patients with Histopathological Positive Frozen Sections

If the histopathological analysis of a frozen section was positive for residual OSCC, a second resection of the compromised area within the former tumor bed was indicated. This was done either within primary surgery or within 21 days after primary surgery.

Frozen sections of 18 patients (5 female and 13 male) still showed tumor residuals. In all of these patients, a second resection within primary surgery was performed to achieve a sufficient safety margin (see Table 8). In 38.89% of these patients (60% of female patients and 30.77% of male patients), a further resection within 21 days after the primary surgery was performed (Table 8).

- Frequency of Frozen Section Preparation for Immediate Histopathological Evaluation

Information about the presence/absence of fresh intraoperatively obtained biopsies analyzed as frozen sections and the histopathological outcome of frozen sections was available for *n* = 157 patients. Analysis of histopathological outcome, in terms of the presence of residual OSCC within the sample, showed a ratio of 17.6% positive frozen sections in patients later diagnosed with recurrence and 11.4% positive frozen sections in patients without recurrences in follow-up (see Table 9). No statistically significant increase in recurrence probability was found for positive frozen sections (*p* = 0.4019).

Patients diagnosed with recurrences more than 1 year after primary surgery were the minority (see Table 10). Recurrence was mostly diagnosed within the first year (16.6% of all patients), whereas only 7.7% were diagnosed with recurrence after the first year, compared to 75.74% of patients w/o recurrence. Distribution according to the T-stage was comparable to the overall distribution. Furthermore, the majority of histopathological positive frozen sections were found in patients with T2 to T4 tumors: T1: 4.17% in T1 tumors, 15.0% in T2 tumors, 20.0% in T3 tumors, and 19.23% in T4 tumors (see Table 10).

- Influence of Frozen Section Outcome on Time Point of Recurrence Diagnosis

An analysis of patients diagnosed with recurrences showed the presence of residual OSCC in frozen sections in 17.4% of patients with recurrence within the first year and 18.2% of patients with recurrence observed more than one year following primary surgery (see Table 11). No statistically significant increase in the probability of early occurrence of recurrence was observed if the frozen section had a positive histological outcome (*p* > 0.99).

- Dependency of Frozen Section Outcome on Recurrence-Free Survival

Patients were analyzed within groups according to follow-up time of up to 12, 12–24, 24–36, and more than 36 months. Clinical data from follow-up of at least 12 months were available for 45 female and 44 male recurrence-free patients (Table 12). Data of recurrence-free patients up to 24 months were available for 15 female and 14 male patients, up to 36 months for 11 female and 5 male patients, and for more than 36 months for 19 female and 25 male patients.

Out of these recurrence-free patients, 16.87% of patients (10% of female patients and 23.26% of male patients) with a follow-up of at least 12 months had histopathological positive frozen sections (see Table 12).

Patients without recurrences observed up to 24 months had in 42.1% of frozen sections OSCC residuals detected. Patients without recurrences observed up to 36 months had 6.7%, and patients with a follow-up of more than 36 months showed 25.0% of OSCC residuals in analyzed frozen sections (see Table 12). Even though up to 62.5% (Table 12, frozen section outcome all male patients with follow-up of 24 months) of analyzed frozen sections were positive with residual OSCC, no recurrence was diagnosed in these patients.

Clinical data from follow-ups of at least 12 months were available for seven female and eight male patients with recurrence (Table 13). Data on recurrence patients up to 24 months were available for one female and one male patient, up to 36 months for one female and two male patients, and for more than 36 months for five female and three male patients.

Distribution of patients according to the time period of follow-up showed that 15.15% (15.6% female, 18.2% male) of all patients with a follow-up of at least 12 months were diagnosed with recurrence within the first year. Out of these patients, 25.0% had histopathological positive frozen sections (see Table 13). Only a minority of patients with follow-up up to 24, 36, or more than 36 months were diagnosed with recurrence after the first year. Out of all these groups, only one patient had a histopathological outcome of frozen sections (Table 13).

#### 3.5.2. Resection Margins

The final histopathology of OSCC resection gives information about the final resection status and the resection margin. This resection margin can further be classified as being clear (>5 mm margin), close (1–5 mm margin), or involved (<1 mm margin) according to the Royal College of Pathologists (RCPath) 1998 [5].

- Distribution of Recurrence and Recurrence-Free Patients According to Resection Margin

Information of final histopathology regarding the definite resection margin was available for *n* = 154 patients, *n* = 46 patients w/recurrence, and *n* = 108 patients w/o recurrence. According to the final histopathology, the resection margin of 14.3% of these patients could be categorized as “clear (>5 mm)”, 50.6% as “close (1–5 mm)”, and 35.1% as “involved (<1 mm)” (see Table 14). The distribution of patients according to diagnosis with/without recurrences was comparable. Patients with recurrence showed the following resection margin distribution: 10% clear, 50% close, and 40% involved. Distribution in patients without recurrence was 15.8% clear, 50.9% close, and 33.3% involved.

Statistical analysis showed that the probability to get recurrence was not statistically significantly different if the resection margin was “involved” instead of “close” (*p* = 0.6921) or “clear” (*p* = 0.3954). Also, no statistically enhanced or reduced probability to get recurrence was found if the resection margin was “close” instead of “clear” (*p* = 0.5796).

- Distribution of Recurrence and Recurrence-Free Patients According to Resection Margin and Therapeutic Approach with or Without Adjuvant Therapy

Of the 22 patients with a resection margin categorized as “clear”, 14 did not receive adjuvant therapy, while 8 underwent adjuvant treatment post-surgery. In the cohort of 14 patients without adjuvant therapy, 2 (14.3%) were later diagnosed with recurrence, whereas recurrence was observed in 2 of the 8 patients (25%) who received adjuvant therapy (Table 15). The probability of recurrence if the resection margin was “clear” and no adjuvant therapy was given was not statistically significant (*p* = 0.6019).

Among the 78 patients with a resection margin categorized as “close”, 36 did not receive adjuvant therapy, while 42 underwent adjuvant treatment post-surgery. Recurrence was observed in 8 of the 36 patients (22.2%) without adjuvant therapy and in 12 of the 42 patients (28.6%) who received adjuvant therapy during follow-up (Table 15). The probability of recurrence in patients with a “close” resection margin who did not receive adjuvant therapy was not statistically significant (*p* = 0.6076).

In total, 54 patients had final histopathology categorized as “involved”, with a nearly equal distribution between those who did not receive adjuvant therapy (28 patients) and those who did (26 patients). Recurrence was observed in 11 of the 28 patients (39.3%) without adjuvant therapy and in 5 of the 26 patients (19.3%) who received adjuvant therapy during follow-up (Table 15). The difference in recurrence rates between patients with an “involved” resection margin who did or did not receive adjuvant therapy was not statistically significant (*p* = 0.141).

## 4. Discussion

Important primary goals in the management of oral squamous cell carcinoma (OSCC) patients are the achievement of local recurrence-free disease and improved overall survival. The results of the present study, however, raise many questions about the true factors responsible for recurrence in OSCC patients. Our overall recurrence rate was 24.3%, and this compares favorably with previous studies [5]. The majority of these recurrences (68.3%) occurred within the first year following the first surgical intervention, underscoring the importance of close postoperative follow-up especially in the first year post-surgery. This was also the opinion of Sasaki et al., who had a recurrence rate of 86.3% in their first year postoperative period and, therefore, advocated for a stringent 2-weekly follow-up with monthly ultrasound in the first year and further CT or MRI investigation if findings are suspicious [15].

From our results, additionally, the stage of the disease did not seem to be indicative in determining whether recurrence would occur. In fact, there were fewer cases of recurrence within the first year of follow-up in T4 tumors when compared to T2 and T3 tumors from our patients’ cohort. This means that the stage of OSCC alone may be of little predictive value in terms of local recurrence and overall survival and that early-stage tumors should be managed as “aggressive” as late stages. This was similar to a previous study that focused on the histological grade of early OSCC cases and reported no predictive value with these grades of tumors in terms of recurrence [16]. Nevertheless, as the Kaplan–Meier survival analysis revealed, a T-stage-dependency was observed for survival in OSCC patients without recurrences, with poorer survival associated with advancing stages, except for T4 tumors, which showed higher survival rates than T3 tumors. Survival was further dependent on the presence of recurrence, as well as on whether recurrences occurred within the first year or later. Patients with recurrences within the first year had significantly lower survival probabilities compared to those without recurrences, with initial T-stage dependency diminishing in the following years. Recurrences occurring after the first year showed better survival probabilities, though the results for this group should be interpreted with caution due to the small sample size.

The highest number of cases with recurrences were found for the locations with the highest OSCC frequency, such as the tongue, floor of mouth, and lower jaw. Our study, like others, reported the tongue as the site most frequently affected by OSCC [9,17]. The floor of the mouth [18,19], the buccal mucosa [20], the lip [21], and the upper and lower jaws [22] had all been previously reported in different studies as the commonest site for OSCC occurrence. These variations may be due to different geographical locations and the prevalent social habits. It is also worth noting that even among our study sample, there was variation in tumor location based on gender: the tongue was the most common site in males, whereas it was the floor of the mouth in females. This gender-based variation has also been noted by other authors [17,23]; however, the reason still remains largely unknown.

As stated earlier, the decision to use adjuvant therapy (radiotherapy (RT) or chemoradiotherapy (CRT)) post-surgery in our center is made by the tumor board expert panel and with the patient’s consent. The panel considers each case individually depending mainly on the stage of the disease at presentation, nodal involvement, extracapsular nodal extension, perineural invasion, intraoperative surgical margin status, and post-surgical histopathological outcome. Interestingly, the patients who received adjuvant therapy and those who did not from our study had comparable outcomes in terms of local recurrence, with no enhanced probability of developing recurrence if adjuvant therapy was not used. A review by Kiyota et al. also showed that RT alone as adjuvant therapy in HNSCC resulted in high local recurrences and distance metastasis, with low 5-year survival, and this was not significantly improved even when a combination CRT was used [13]. Similarly, a large multicenter study reported a significantly reduced quality of life for irradiated patients with seemingly no influence on the overall survival in early-stage OSCC and oropharyngeal cancers [12]. In the same vein, two other studies that focused on OSCC patients with intermediate risk treated post-surgery with RT did not yield an improved overall survival when compared to the control groups [12]. These studies suggest that it may be advantageous to withhold RT from such groups of patients without compromising survival, bearing in mind the adverse effects of RT and the subsequent poor quality of life it could induce. All these controversies regarding the use of adjuvant therapy in OSCC patients highlight the need for further studies to better stratify which patient groups specifically require RT or CRT.

On the other hand, the dilemma in the management of tongue and floor of the mouth OSCC is ever-present. This is due to the debilitating nature of the surgery in those locations and the attendant poor quality of life, especially in terms of speech, deglutition, and overall nutrition. Our study revealed that the tongue and the floor of the mouth had the highest recurrence rate than all the other sites and these recurrences were irrespective of the stage of the disease and the treatment modalities applied. This raises the question of whether tumors of the tongue and floor of the mouth would achieve a better outcome with definitive chemoradiotherapy (CRT) alone, or with a more sophisticated treatment regimen prioritizing C(R)T as a neoadjuvant approach before surgery. It has been well established that primary RT is a recommended curative treatment in early-stage small oral cancers including tongue lesions [24]. However, in advanced stages of oral cancers (stages III and IV), there is no consensus on the best management approach as different outcomes have been documented over the years following different treatment modalities. Previous reports have indicated no statistically significant differences in locoregional control, overall survival, or even disease-free survival in advanced resectable OSCC treated either with primary RT or when used as adjuvant [25,26]. This was, however, not the case in a recent meta-analysis by Forner et al., which found that primary RT or CRT as a definitive treatment of OSCC remains inferior to primary surgical treatment with a significantly increased risk of death in early-stage OSCC [27]. In a recently published approach from our center applying neoadjuvant chemoradiotherapy (nCRT) followed by surgery (INVERT trial), it was shown that this regimen is feasible for locally advanced oral cavity cancer (LA-OCC) and achieves high response rates, with 41% of patients showing a complete pathological response [14]. Given these results, it is reasonable to assume that such a regimen might represent a superior approach for the particularly challenging tumors of the tongue and floor of the mouth. Preliminary findings from this trial further suggest that diffusion-weighted MRI signal changes and specific tumor-infiltrating immune cells may serve as predictive biomarkers for treatment response, which could further improve the patient-specific treatment planning of such challenging cases. However, further investigation in larger trials is needed to confirm these findings [14].

During surgery for OSCC in our center, if histopathological analysis of frozen section margins was positive for residual OSCC, a second resection of the compromised area within the former tumor bed was indicated. This was done either during primary surgery or within 21 days after primary surgery with the aim of achieving R0 status. From our results, the majority of the positive histopathological frozen section margins were found in T2 to T4 patients. Among the patients with recurrences following primary surgery, there was no statistically significant difference between patients who had positive frozen sections and those who did not. On the other hand, for patients without recurrences who were observed up to 24 months, 42.1% of them had positive frozen sections, and this was even significantly up to 62.5% when considering male patients alone.

These findings agree with some earlier reports that suggested that survival and loco-regional recurrences were similar in patients with negative frozen section margins and those with positive margins that needed re-resection [28,29]. On the contrary, Szewczyk et al. demonstrated in their study that positive intraoperative frozen margins were significantly associated with local recurrence and, therefore, may portend a more aggressive variant of OSCC for which an initial R0 margin could not be achieved [4]. They also proved through multivariate analysis that a positive frozen margin represented the only independent adverse factor and, therefore, should be considered while planning further treatment. This was also the opinion of Guillemaud et al., who concluded that the initial positive frozen section alone represented a poor overall OSCC prognosis despite the final margin outcome [30].

It therefore means that there is no consensus on how these patients with initial positive fresh frozen margins should be subsequently managed. This disagreement in findings may be because of the limitations of the reviewed studies including ours. It should be noted that these studies were all retrospective reviews and, as observed by Szewczyk et al., the direct impact of positive/negative fresh frozen section margins and other factors examined could have been diminished by the high percentage of patients who received adjuvant therapy administration [4]. Even though re-resection may appear to be non-inferior to immediate resection depending on the trial considered, the avoidance of a second surgery should clearly be prioritized, taking into account quality of life and economic aspects alone. Moreover, considering other studies that show an increased risk associated with later re-resection, the risks may be too high, further supporting the use of intraoperative frozen section control. In the final histopathology, patients with recurrence had resection margins of 10% clear, 50% close, and 40% involved. In contrast, patients without recurrence had 15.8% clear, 50.9% close, and 33.3% involved. Further analysis showed, interestingly, no statistically significant difference in recurrence between involved and close margins (*p* = 0.6921) or between close and clear margins (*p* = 0.5796), even though the final resection margin is considered one of the important indicators to guide the choice of further treatment of OSCC patients. However, there is no unanimous agreement in the literature as to whether the histopathologic margin of ≤5 mm (close) should be subjected to adjuvant therapy postoperatively, even when there are no other risk factors [31,32,33]. Brinkman et al. suggested a 3 mm demarcation between close and clear margins instead of the previous 5 mm advocated by the RCPath, claiming that this cut-off has a prognostic significance [6]. Similarly, Wong et al., while suggesting a 2 mm cut-off margin based on their own study, recommended that other prognostic factors should be considered in the decision for further treatment [31]. It is interesting to note that among our patients’ cohort, we did not find any statistically significant difference in terms of local recurrence for OSCC patients with involved, close, or clear margins when no adjuvant therapy was given. The question therefore arises as to whether we are indeed overtreating the patients by trying to achieve a clear margin bearing in mind the attendant morbidity when indeed similar outcomes could be reached irrespective of the final histopathologic margin status, as demonstrated by our study.

### Limitation of the Study

First, the retrospective nature of the trial introduces the potential for selection bias and limits the ability to establish causal relationships. The data were extracted from patient records, which, unlike in prospective studies, lack a standardized and comprehensive depth of documentation. Additionally, data were not available for all endpoints, which may affect the completeness of the analysis. Furthermore, the data are derived from a single-center cohort, limiting the generalizability of the findings. As such, the conclusions drawn from this study are specific to the institution and need to be validated or challenged through comparison with similar data from other centers before any broader applicability can be confirmed. The retrospective design also precluded the possibility of conducting quality-of-life assessments, which could have provided valuable insights into outcomes from the patients’ perspective.

## 5. Conclusions

The OSCC of the tongue had the highest rate of recurrence from our study, and most of the recurrences occurred within the first year of primary surgical intervention with the stage of the disease not playing any significant role. This raises the question as to the place and relevance of radical surgery in OSCC management with the resultant postoperative morbidity and poor quality of life, especially in locations like the tongue and the floor of the mouth. Should tumors in these locations achieve better outcomes with chemoradiotherapy (CRT) alone, without initial surgery, or should treatment follow the approach outlined in the INVERT trial [14], with nCRT followed by surgery? This question highlights the need for further studies to explore the potential of non-surgical management for tongue and floor-of-mouth OSCC and to clarify the role of CRT in this context.

The frozen section margins and the final histopathologic margins did not influence the occurrence of recurrence either. Additionally, there was a comparable outcome in terms of recurrence and overall survival for patients who received adjuvant therapy and those who did not. These findings clearly show that achieving recurrence-free disease in OSCC cases may not be entirely dependent on the technique of surgical management and the subsequent adjuvant therapy. Close attention should be paid to the nature of the tumor in terms of its biology from the first biopsy and treatment planned on a case-by-case basis in order to achieve optimal results for each individual patient.

## Figures and Tables

**Table 1:** Distribution of patients (total and female/male) according to tumor localization.

Tumor Localization	Number of Cases (Female/Male)
Lip	**3** (1/2)
Lower jaw	**27** (12/15)
Upper jaw	**16** (11/5)
Tongue	**56** (32/24)
Floor of mouth	**51** (19/32)
Palate	**8** (5/3)
Buccal mucosa	**8** (5/3)

**Table 2:** Distribution of all analyzed patients according to T-stage (n_total_ = 169 patients).

Tumor Stage	Female	Male	Total
T1	29	22	51
T2	20	31	51
T3	21	15	36
T4	15	16	31
Total all T-stages	85	84	169

**Table 3:** Distribution of patients with/without recurrences according to gender. *n* = 169.

Sex	w/Recurrence	w/o Recurrence
Male	21	63
Female	20	65

**Table 4:** Distribution of patients with/without recurrences according to gender and year of recurrence diagnosis.

	Within the First Year		Within the Second Year		Within the Third Year	
	w/Recurrence	w/o Recurrence	w/Recurrence	w/o Recurrence	w/Recurrence	w/o Recurrence
Male	15	14	1	14	2	5
Female	13	17	1	15	1	11
*p*-value	0.6058		>0.99		0.5232	

**Table 5:** Distribution of all analyzed patients (n_total_ = 169 patients) w/o recurrences, w/recurrences within the first year, and more than one year post-surgery according to T-stage.

	Recurrence					
	w/o Recurrence		w/Recurrence within the First Year		w/Recurrence >First Year	
	F	M	F	M	F	M
T1 total	23	18	3	2	3	2
T2 total	12	21	5	7	3	3
T3 total	18	11	3	3	0	1
T4 total	12	13	2	3	1	0
Total all T-stages	65	63	13	15	7	6

**Table 6:** Distribution of patients with/without recurrences according to additional adjuvant therapy.

Adjuvant Therapy	w/Recurrence	w/o Recurrence
w/o adjuvant therapy	22	67
w/adjuvant therapy	19	60

**Table 7:** Distribution of patients with/without recurrences according to the type of adjuvant therapy.

Type of Adjuvant Therapy	w/Recurrence	w/o Recurrence
CRT	8	30
Radiotherapy	11	30

**Table 8:** Distribution of all analyzed patients with histopathological positive frozen sections and distribution of second resections, according to T-stage and sex.

	Number of Patients with Positive Frozen Sections		2nd Resection Within Primary Surgery		Further Resection Within 21 Days Post Primary Surgery	
	F	M	F	M	F	M
T1 total	1	1	1	1	0	1
T2 total	1	5	1	5	1	2
T3 total	1	4	1	4	1	1
T4 total	2	3	2	3	1	0
Total all T-stages	5	13	5	13	3	4

**Table 9:** Distribution of patients with/without recurrences according to the histopathological outcome of frozen section analysis during primary surgery.

Frozen Sections (FS) Outcome	w/Recurrence	w/o Recurrence
Ratio of FS taken (FS/total)	97.56% (40 of 41)	91.41% (117 of 128)
FS positive	6	12
FS negative	34	105

**Table 10:** Distribution of all analyzed patients without recurrences, with recurrences within the first year, and more than one year post-surgery regarding the evaluation of frozen sections (FS) and outcomes according to T-stage. (“+” = positive FS with residual OSCC; “−“ = negative FS).

	Recurrence					
	w/o Recurrence		w/Recurrence within the First Year		w/Recurrence >First Year	
	F	M	F	M	F	M
T1 total	23	18	3	2	3	2
FS taken in primary surgery	23	17	3	2	3	2
FS outcome	0+/23−	0+/17−	1+/2−	1+/1−	0+/3−	0+/2−
T2 total	12	21	5	7	3	3
FS taken in primary surgery	9	19	5	7	3	3
FS outcome	0+/9−	4+/15−	0+/5−	0+/7−	1+/2−	1+/2−
T3 total	18	11	3	3	0	1
FS taken in primary surgery	15	9	2	3	0	1
FS outcome	1+/14−	3+/6−	0+/2−	1+/2−		0+/1−
T4 total	12	13	2	3	1	0
FS taken in primary surgery	12	13	2	3	1	0
FS outcome	2+/10−	2+/11−	0+/2−	1+/2−	0+/1−	
Total all T-stages	65	63	13	15	7	6
FS taken in primary surgery	59	58	12	15	7	6
FS outcome	3+/56−	9+/49−	1+/11−	3+/12−	1+/6−	1+/5−

**Table 11:** Distribution of patients with recurrence within the first and later than the first year according to the histopathological outcome of frozen section analysis during primary surgery.

Frozen Sections (FS) Outcome	w/Recurrence within the First Year	w/Recurrence Post-First Year
FS positive	4	2
FS negative	23	11

**Table 12:** Patients distribution according to recurrence-free survival, up to 12, 24, 36, and >36 months post-surgery regarding the evaluation of frozen sections (FS) and outcome according to T-stage. (“+” = positive FS with residual OSCC; “−” = negative FS).

	Recurrence-Free Survival According to Follow-Up							
	Follow-Up up to 12 Months		Follow-Up up to 24 Months		Follow-Up up to 36 Months		Follow-Up >36 Months	
	F	M	F	M	F	M	F	M
T1 total	21	12	5	1	4	2	12	9
FS taken in primary surgery	21	12	5	1	4	2	12	9
FS outcome	0+/21−	0+/12−	0+/5−	0+/1−	0+/4−	0+/2−	0+/12−	0+/9−
T2 total	8	15	2	4	1	1	5	10
FS taken in primary surgery	7	14	1	3	1	1	5	10
FS outcome	1+/6−	7+/7−	1+/1−	3+/0−	0+/1−	0+/1−	0+/5−	4+/6−
T3 total	6	6	1	3	4	1	1	2
FS taken in primary surgery	6	6	1	3	4	1	1	2
FS outcome	1+/5−	1+/5−	1+/0−	1+/2−	0+/4−	0+/1−	0+/1−	0+/2−
T4 total	10	11	7	6	2	1	1	4
FS taken in primary surgery	10	11	7	6	2	1	1	4
FS outcome	2+/8−	2+/9−	1+/6−	1+/5−	1+/1−	0+/1−	0+/1−	1+/3−
Total all T-stages	45	44	15	14	11	5	19	25
FS taken in primary surgery	44	43	14	13	11	5	19	25
FS outcome	4+/40−	10+/33−	3+/11−	5+/8−	1+/10−	0+/5−	0+/0−	5+/20−

**Table 13:** Distribution of patients with follow-up of at least 12 months with recurrences within the first year post-surgery, and those with recurrences after the first year (in follow-up up to 24, 36, and >36 months post-surgery) regarding the evaluation of frozen sections (FS) and outcome according to T-stage. (“+” = positive FS with residual OSCC; “−” = negative FS).

	Recurrence within the First Year		Recurrence > 1 Year Post-Surgery					
	Follow-Up Up to 12 Months		Follow-Up Up to 24 Months		Follow-Up Up to 36 Months		Follow-Up >36 Months	
	F	M	F	M	F	M	F	M
T1 total	3	2	0	0	0	1	3	1
FS taken in primary surgery	3	2				1	3	1
FS outcome	1+/2−	1+/1−				0+/1−	0+/3−	0+/1−
T2 total	4	4	0	0	1	1	2	2
FS taken in primary surgery	4	4			1	1	2	2
FS outcome	0+/4−	0+/4−			1+0−	1+/0−	0+/2−	0+/2−
T3 total	0	1	0	1	0	0	0	0
FS taken in primary surgery		1		1				
FS outcome		0+/1−		0+/1−				
T4 total	0	1	1	0	0	0	0	0
FS taken in primary surgery		1	1					
FS outcome		1+/1−	0+/1−					
Total all T-stages	7	8	1	1	1	2	5	3
FS taken in primary surgery	7	8	1	1	1	2	5	3
FS outcome	1+/6−	2+/6−	0+/1−	0+/1−	1+/0−	1+/1−	0+/5−	0+/3−

**Table 14:** Distribution of patients with/without recurrences (n_total_ = 154).

Resection Margin	w/Recurrence	w/o Recurrence	Total
Clear (>5 mm)	4	18	22
Close (1–5 mm)	20	58	78
Involved (<1 mm)	16	38	54

**Table 15:** Distribution of patients with/without recurrences according to additional adjuvant therapy, categorized according to the resection margin in final histopathology (n_total_ = 154).

Therapeutic Approach	w/Recurrence	w/o Recurrence	Total
clear (>5 mm)			
w/o adjuvant therapy	2	12	14
w/adjuvant therapy	2	6	8
close (1–5 mm)			
w/o adjuvant therapy	8	28	36
w/adjuvant therapy	12	30	42
involved (<1 mm)			
w/o adjuvant therapy	11	17	28
w/adjuvant therapy	5	21	26

## Data Availability

The data presented in this study are available in this article.
