# Correction: Ghanaati et al. Cancer Recurrence in Operated Primary Oral Squamous Cell Carcinoma Patients Seems to Be Independent of the Currently Available Postoperative Therapeutic Approach: A Retrospective Clinical Study. *Curr. Oncol.* 2025, *32*, 208

**DOI:** 10.3390/curroncol33020073

**Published:** 2026-01-27

**Authors:** Shahram Ghanaati, Samuel Ebele Udeabor, Anne Winter, Robert Sader, Anja Heselich

**Affiliations:** 1 Department of Oral, Cranio-Maxillofacial, and Facial Plastic Surgery, Johann Wolfgang Goethe University, 60596 Frankfurt am Main, Germany; seudeabor@kku.edu.sa (S.E.U.); anne.winter@unimedizin-ffm.de (A.W.);; 2 Department of Oral and Maxillofacial Surgery, College of Dentistry, King Khalid University, Abha 61421, Saudi Arabia

**Text Correction**

There was an error in the original publication [1]. The given ratios in percent were the ratios of correlation between the outcomes, but not related to the overall patient cohort as originally intended. To omit any misunderstanding, corrections have been made to the following chapters:

3.2.3. Recurrences

Overall Recurrence Distribution and Occurrence of Recurrences Within Follow-Up

“Patients’ follow-up revealed diagnosed recurrence in 25% of female patients and 23.5% of male patients (see Table 3).”

Recurrence Distribution According to Tumor Localization

“The ratio of cases w/recurrences showed the following distribution: 30.6% for tumors in the tongue (17 out of 56 cases), 17.6% for tumors in the floor of the mouth (9 out of 51 cases), 29.6% for tumors in the lower jaw (8 out of 27 cases), 12.5% for tumors in the upper jaw (2 out of 16 cases), 25% for tumors in the buccal mucosa (2 out of 8 cases), and 37.5% for tumors at the palate (3 out of 8 cases).”

3.4. Adjuvant Therapy Regime in OSCC Patients with and Without Recurrences

“Sub-analysis of patients who received adjuvant therapy showed that 21.05% of patients undergoing chemoradiotherapy (CRT) and 26.83% of patients receiving radiotherapy developed recurrence during follow-up (see Table 7).”

3.5. Histopathological Outcomes

Frequency of Frozen Section Preparation for Immediate Histopathological Evaluation

“Analysis of histopathological outcome, in terms of the presence of residual OSCC within the sample, showed a ratio of 15.0% positive frozen sections in patients later diagnosed with recurrence and 10.3% positive frozen sections in patients without recurrences in follow-up (see Table 9).”

“Furthermore, the majority of histopathological-positive frozen sections were found in patients with T2 to T4 tumors: T1: 4.0% in T1 tumors, 13.0% in T2 tumors, 16.7% in T3 tumors, and 16.1% in T4 tumors (see Table 10).”

Influence of Frozen Section Outcome on Time Point of Recurrence Diagnosis

“An analysis of patients diagnosed with recurrences showed the presence of residual OSCC in frozen sections in 14.8% of patients with recurrence within the first year and 15.4% of patients with recurrence observed more than one year following primary surgery (see Table 11).”

Dependency of Frozen Section Outcome on Recurrence-Free Survival

“Out of these recurrence-free patients, 16.1% of patients (9.1% of female patients and 23.26% of male patients) with a follow-up of at least 12 months had histopathological-positive frozen sections (see Table 12).

Patients without recurrences observed up to 24 months had in 29.6% of frozen sections OSCC residuals detected. Patients without recurrences observed up to 36 months had 6.25%, and patients with a follow-up of more than 36 months showed 20.0% of OSCC residuals in analyzed frozen sections (see Table 12). Even though up to 38.5% (Table 12, frozen section outcome all male patients with follow-up of 24 months) of analyzed frozen sections were positive with residual OSCC, no recurrence was diagnosed in these patients.”

“Distribution of patients according to the time period of follow-up showed that 14.4% (13.5% female, 15.4% male) of all patients with a follow-up of at least 12 months were diagnosed with recurrence within the first year. Out of these patients, 20.0% had histopathological-positive frozen sections (see Table 13).”

The authors state that the scientific conclusions are unaffected. This correction was approved by the Academic Editor. The original publication has also been updated.
